# Medical liability, defensive medicine and professional insurance in otolaryngology

**DOI:** 10.1186/s13104-015-1318-2

**Published:** 2015-08-11

**Authors:** Sergio Motta, Domenico Testa, Ugo Cesari, Giuseppe Quaremba, Gaetano Motta

**Affiliations:** Department of Neurosciences, Institute of Otorhinolaryngology, Ateneo “Federico II”, Via S. Pansini no. 5, 80100 Naples, Italy; Department of Otorhinolaryngology, II University of Naples, Via S. Pansini no 5, 80100 Naples, Italy; Department of Forensic Medicine, University “Federico II”, Via S. Pansini no 5, 80100 Naples, Italy; Via Stazio no 8, 80123 Naples, Italy

**Keywords:** Medical malpractice, Civil liability, Professional liability insurance, Defensive medicine

## Abstract

**Background:**

This study aims at verifying relationships between the perception of medico-legal risks involved in the professional activity of Italian otolaryngologists, defensive medical behaviour and their understanding of professional liability insurance in matters of civil liability. One hundred specialists replied to a questionnaire pertaining to the psychological impact of medico-legal issues and to specific queries regarding insurance coverage, either privately stipulated or provided by the employer. Statistic analysis was carried out by χ^2^ test and ANOVA multiple variance regression test, assuming P = 0.05 as the value of minimum statistical significance.

**Results:**

It was found that in 50 % of cases the behaviour of the doctor towards the patient had been decidedly influenced by concerns over medico-legal implications. In 29 % of the sample these concerns had “often to always” influenced the choice of diagnostic procedures or treatment options, in order to safeguard themselves in case of legal dispute. The data obtained showed a statistically significant correlation between the level of concern (regarding potential medico-legal disputes) experienced by specialists on the one hand and variations in the doctor/patient relationship (P < 0.05) and the choice of defensive medical procedures (P < 0.05) on the other. Furthermore, the perception of the medico-legal problem was statistically related to the absence or poor knowledge of some insurance clauses, regarding posthumous coverage (72 %), informed written consent (89 %), and the coverage provided by the healthcare centre where the specialist is employed (32 %) (P < 0.05).

**Conclusions:**

The results of this study indicate the necessity for a greater awareness of the actual guarantees provided by the insurance policy stipulated by specialists, to avoid inadequate coverage in the case of medico-legal disputes.

**Electronic supplementary material:**

The online version of this article (doi:10.1186/s13104-015-1318-2) contains supplementary material, which is available to authorized users.

## Background

For more than 30 years the question of medical malpractice and consequent civil liability has been debated in judicial and healthcare circles in all nations with high level healthcare [1].

Defensive medicine is defined as the ordering of tests and procedures (positive defensive medicine), or the avoidance of high-risk patients or procedures (negative defensive medicine), primarily to reduce exposure to malpractice liability [2].

This behaviour has become deeply ingrained in many physicians’ practices [3] resulting in a difficult to quantify “unconscious” defensive medicine [4]. Few data are available regarding “defensive” procedures in otolaryngology and are restricted to very specific aspects of the field [5]. The present investigation aims at clarifying the most interesting aspects of the issue and above all, establishing the actual relationships between medico-legal risk perception and the professional activity of otolaryngologists in Italy, defensive medical behaviour [6, 7] and the degree of understanding that specialists actually have of the insurance policies stipulated by them [8, 9].

## Methods

The present study was considered exempt from ethical approval, as stated by the Ethic Committee of the University of Naples “Federico II”.

One hundred out of 858 (11.6 %, mean age: 51.8; SD 10.6) specialists from the Italian Society of Otolaryngology (SIO) answered an email questionnaire (Additional file 1) regarding the psychological impact of medico-legal issues and specific queries concerning insurance cover, whether privately stipulated or provided by the employer. The completion and transmission of the questionnaire were considered to imply a written informed consent by all the respondent specialists.

The personal and professional data of our sample are shown in Table 1.

**Table 1:** Personal and professional data of the specialists interviewed

Number of respondents	100
Average age (SD)	51.8 years (±10.6)
Sex	73 Men
	17 Women
Number of years as a specialist	20 % up to 10 years of professional activity
	77 % over 10 years of professional activity
	3 % no answer
Category	52 % hospital
	18 % university
	7 % other healthcare centres
	18 % other
	2 % no answer
Surgical activity	59 % yes, not cosmetic
	18 % surgical and cosmetic
	23 % no answer or no surgical activity
Qualification	76 % medical consultant of various levels
	24 % no answer or no medical consultant qualification

Statistical analysis was carried out by χ^2^ test and ANOVA multiple variance regression test, assuming P = 0.05 as the minimum value of statistical significance.

## Results

### Perception of medico-legal problem, physicians-patients relation and defensive procedures

**Fig. 1:**
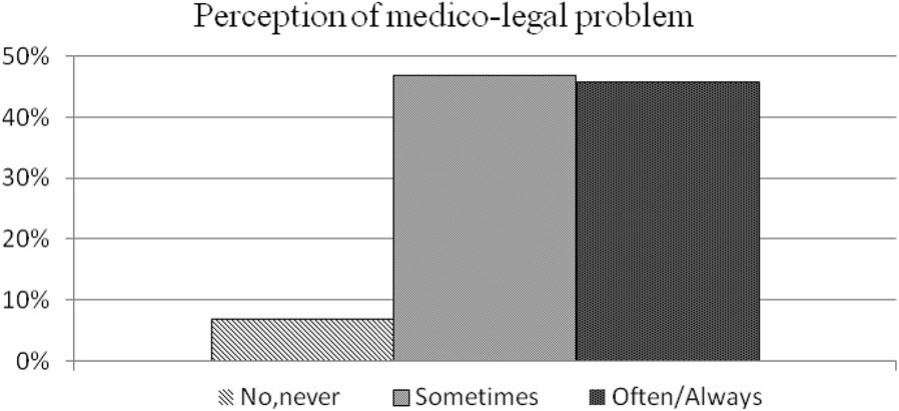
Answers to the query (100 subjects): “Over the last 2–3 years, during your professional work, have you considered the possibility of medico-legal repercussions that could result as a consequence of a diagnostic/therapeutic procedure?”.

**Fig. 2:**
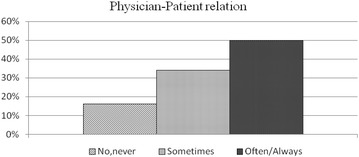
Answers to the query (100 subjects): “Over the last 2 or 3 years, has the concern for medico-legal consequences determined a significant variation in your doctor/patient relations, including communication and information?”.

**Fig. 3:**
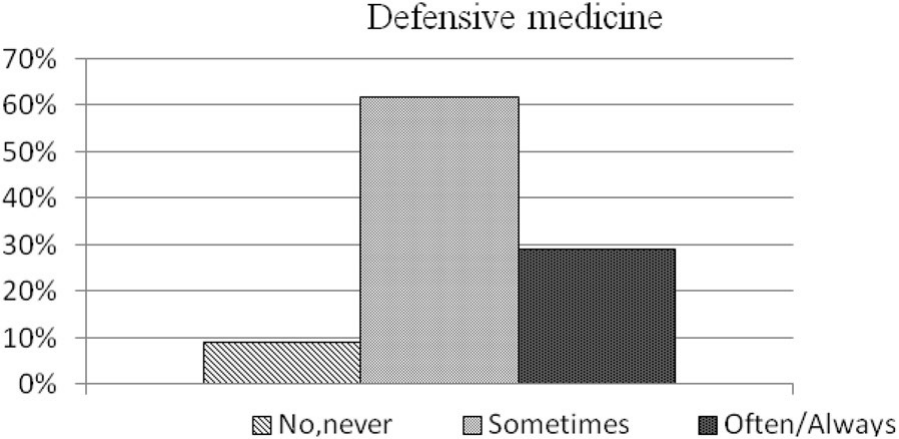
Answers to the query (100 subjects): “Over the last 2 or 3 years has the concern for medico-legal consequences determined a significant variation in the choice of diagnostic procedures or treatments that you would not have otherwise prescribed as a precautionary measure against possible medico-legal disputes?”.

Forty-six percent of the specialists interviewed declared they “often to always” considered the possibility of medico-legal repercussions inherent in the performance of any diagnostic or therapeutic procedure (Fig. 1). In 50 % of the cases, concern over medico-legal implications had had a decisive influence (Fig. 2) on their relations with patients (including communication and information). In 29 % of the sample these concerns had “often to always” influenced the choice of diagnostic procedures or clinical paths prescribed, to safeguard against potential legal dispute (Fig. 3).

Statistical analysis showed a significant correlation between the degree of concern felt by specialists, with regards to potential medico-legal disputes and variations in the doctor/patient relationship (P < 0.05) on the one hand, and the choice of defensive medical procedures (P < 0.05) on the other.

A tendency to a statistically significant relationship between surgical activity and the adoption of defensive type procedures (P = 0.06) was found in those specialists who had stipulated a private contract for professional liability (82 cases).

### Knowledge of insurance clauses

**Fig. 4:**
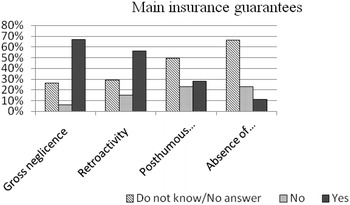
Answers to the query (82 subjects):”Does your private insurance policy provide: (a) cover in the case of gross negligence; (b) retroactive coverage; (c) posthumous guarantee; (d) coverage in the case of the absence of written informed consent?”.

Concern over potential medico-legal repercussions was statistically related to the absence or the scarce to poor knowledge of the fine print of insurance policies (Fig. 4), with reference to posthumous coverage (72 %) and to informed written consent (89 %), as well as the coverage (32 %) provided by the employer (P < 0.05).

No statistically significant relationship was found between other personal and professional details of the specialists and the level of general concern level of comprehension of the insurance clauses.

## Discussion

The results of this survey demonstrate that concern over potential legal action for presumed professional malpractice is widespread among the majority of the otolaryngologists interviewed, confirming similar reports in literature regarding other medical disciplines [3].

A greater level of concern over potential medico-legal disputes was correlated to a significant variation in the doctor/patient relationship, a more frequent use of defensive procedures and to uncertainty regarding the guarantees offered by professional insurance coverage.

Analysis of the results show widespread difficulty in the of insurance clauses, which often refer to new and more ambiguous types of contracts causing doubts to arise about the real value of the insurance guarantees and thus cause significant repercussions on the health care professional activity.

It is also necessary to point out some limitations of our study, including the low number of specialists who responded to the questionnaire compared to the reference sample; it should be added, however, that no statistically significant difference was found in relation to age between the study group and those who did not respond to the questionnaire. It is likely, however, that above all otolaryngologists particularly interested in the medical-legal issues responded to the questionnaire with a higher percentage of responses indicating greater concern regarding professional malpractice suits. Data published in Italy by insurance companies show that the majority of specialists who stipulated a professional liability policy do not perform any surgical activity (ratio of 1–4 between surgeons and physicians), unlike the findings of our survey, in which the majority of the respondents stated that they practiced surgery.

A considerable increase in the adoption of defensive type procedures was observed in the specialists interviewed who practiced surgical activity. Taking this into consideration, the insufficient degree of knowledge of insurance clauses by many specialists in the present study is even more surprising. This low grade of knowledge is confirmed in other disciplines too [10], probably also in relation to the difficulty of interpretation of insurance contracts.

These data raise serious concern regarding the guarantees provided by insurance companies and also show that very often specialists do not possess a clear understanding of the limits of the insurance policy they have stipulated.

In the present national and international climate, which has seen a progressive worsening of the legal position of the healthcare professional [11, 12], the results call attention to the need for greater awareness on behalf of the specialists of the actual guarantees provided by their insurance policy regarding civil liability, in order to avoid the risk of insufficient insurance coverage in the case of legal dispute for medical malpractice.

## Additional file

Additional file 1. Questionnaire employed in the survey.
