# Supplementary material for: Medical liability, defensive medicine and professional insurance in otolaryngology
Source: BMC Res Notes. 2015 Aug 11;8:343. doi: 10.1186/s13104-015-1318-2 (PMC4532248; doi:10.1186/s13104-015-1318-2)
Supplement: Additional file 1. — Questionnaire employed in the survey. [file 13104_2015_1318_MOESM1_ESM.docx]

1) Over the last 2-3 years, during your professional activity, have you considered the possibility of medico-legal repercussions that could result as a consequence of a diagnostic/therapeutic procedure?

2) Over the last 2-3 years, has the concern for medico-legal consequences determined a significant variation in your doctor/patient relation, including communication and information?

3) Over the last 2-3 years, has the concern for medico-legal consequences determined a significant variation in the choice of diagnostic procedures or treatments that you would not have otherwise prescribed as a precautionary measure against possible medico-legal disputes?

4) Does your private insurance provide a coverage for any damage occurring during the period of activity of the policy (“Loss Occurrence” clause)?

5) Does your private insurance provide a coverage for any claim for compensation addressed to you during the period of activity of the insurance policy (“Claims Made” clause)?

6) Does your private insurance policy provide a coverage in the case of gross negligence?

7) Does your private insurance policy provide a retroactive coverage?

8) Does your private insurance policy provide a posthumous guarantee?

9) Does your private insurance policy provide a coverage in the case of the absence of written informed consent?

Framework of the questionnaire employed in the survey. The multiple-choice options, questions related to duration of the guarantees and to knowledge of public insurance coverage, if any, are not shown for brevity.
